# Synthesis and Properties of Carbon Microspheres from Waste Office Paper

**DOI:** 10.3390/molecules28155756

**Published:** 2023-07-30

**Authors:** Mannan Yang, Jian Su, Changqing Fang, Youliang Cheng, Yangyang Li, Yubo Yan, Wanqing Lei

**Affiliations:** 1 School of Mechanical and Precision Instrument Engineering, Xi’an University of Technology, Xi’an 710048, China; ymannan@163.com; 2 Faculty of Printing, Packaging Engineering and Digital Media Technology, Xi’an University of Technology, Xi’an 710054, China; sujian@xaut.edu.cn (J.S.); chengyouliang@xaut.edu.cn (Y.C.); yangyy_er@163.com (Y.L.); yyb3406@163.com (Y.Y.); lwq900529@163.com (W.L.)

**Keywords:** carbon microsphere, waste office paper, hydrothermal, electrochemical performance

## Abstract

As a kind of biomass resource, waste office paper can be used as a carbon precursor to prepare carbon materials. In this work, carbon microspheres with regular shape, uniform particle size and high carbon content were successfully prepared from waste office paper via a hydrothermal synthesis method with sulfuric acid as the catalyst. The effects of reaction temperature and sulfuric acid dosage on the morphology of the carbon microspheres were studied. The formation mechanism of the carbon microspheres was investigated by analyzing the structure and composition of the products. The results show that the hydrolysis of cellulose in waste paper under hydrothermal conditions was the key for the formation of carbon microspheres. The temperature of hydrothermal reaction and the use of sulfuric acid can affect the morphology of carbon microspheres. The carbon microspheres synthesized at 210 °C with 10 mL sulfuric acid have the best surface morphology, with uniform particle size and higher dispersion. Cyclic voltammetry and electrochemical impedance spectroscopy show that the carbon microspheres have good capacitance performance and can be used in capacitors. This study provides a low-cost precursor for carbon microspheres as well as a new method for the recycle of waste paper.

## 1. Introduction

Carbon microspheres are an important member of the carbon materials. Due to their excellent chemical stability, high thermal conductivity and excellent heat resistance, carbon microspheres have good application prospects in the fields of magnetoelectricity, optics, medicine, etc. [1,2]. The main synthesis methods for carbon microspheres include the arc discharge method, carbonized method, chemical vapor deposition, hydrophobic precipitation, high-temperature pyrolysis, hydrothermal synthesis method, etc. [3,4,5,6]. Wang et al. [7] prepared hard carbon with perfect spherical morphology, controllable monodisperse particle size and smooth surface via the hydrothermal synthesis method. The hydrothermal synthesis method can be used to prepare carbon materials under lower reaction temperature (160−350 °C) from biomass using water as the solvent. Moreover, the hydrothermal synthesis method has the advantage of simple operation, low cost and mild reaction process. The abundant functional groups on the surface of carbon materials prepared using the hydrothermal synthesis method makes the materials have good hydrophilicity. It is an ideal method for preparing carbon microspheres with simple operation and high purity.

Biomass is currently considered to be a raw material with great potential because it is a renewable resource with characteristics of wide distribution and easy access [8]. In recent years, due to the depletion and overexploitation of non-renewable natural resources, the development of functional materials from renewable resources, such as biomass, has become crucial.

Biomass (such as glucose, sucrose, fructose, starch, cellulose and rubber wood) [9,10,11,12,13] and other raw materials such as benzene, toluene, acetylene and intermediate asphalt extracted from coal or petroleum [14,15,16,17] have been used as carbon precursors to prepare carbon microspheres with uniform particle size and high dispersion via hydrothermal synthesis method. Sun et al. [18] prepared colloidal carbon microspheres with particle size of 150−1500 nm via hydrothermal reaction at 160–200 °C with glucose as the raw material. The synthesized carbon microspheres were rich in hydrophilic functional groups such as hydroxyl and carbonyl and had good dispersion in water and ethanol. Sevilla et al. [19] prepared a kind of functionalized carbonaceous material (hydrochar) by using the hydrothermal carbonization (250 °C) of eucalyptus sawdust and barley straw. However, this product basically maintained the original microstructure of the raw material and only sporadic spherical products were formed. Sun et al. [20] prepared carbon sphere-CdS/g-C_3_N_4_ composite from corn stover via the hydrothermal method. The straw cannot be directly used in hydrothermal reactions, and a series of processes such as drying, grinding, sodium hydroxide solution treatment, sulfuric acid solution treatment, etc., were carried out to obtain the cellulose for hydrothermal reactions from straw. It was found that carbon microspheres can be synthesized from monosaccharides, disaccharides, etc. which were produced by deep processing of biomass, but it is difficult to directly use the hydrothermal method to synthesize carbon microspheres from untreated biomass raw materials.

The main components of most biomass materials are cellulose, hemicellulose and lignin, the content of which varies between materials from different sources. Paper is a widely used biomass material product in packaging, office, books, newspapers, etc. [21,22]. It is worth noting that most of the lignin has been removed in the pulp manufacturing process, so the content of cellulose in paper is relatively high. Therefore, the process of synthesizing carbon microspheres from waste paper is simpler than that for biomass, and waste paper is a good raw material for preparing carbon microspheres [23,24,25,26]. More importantly, the cost of waste paper is low, and the recycling of waste paper can also reduce environmental pollution, turn waste into valuable materials and promote the development of a circular economy.

In this study, carbon microspheres were synthesized from waste office paper by controlling hydrothermal hydrolysis conditions, that is, the effects of sulfuric acid concentration and hydrothermal reaction temperature on the morphology of the synthesized carbon microspheres. The results and the formation mechanism of carbon microspheres were analyzed. The electrochemical properties of the synthesized carbon microspheres were also evaluated.

## 2. Results and Discussion

### 2.1. Morphology and Microstructure of Carbon Microspheres

**Figure 1:**
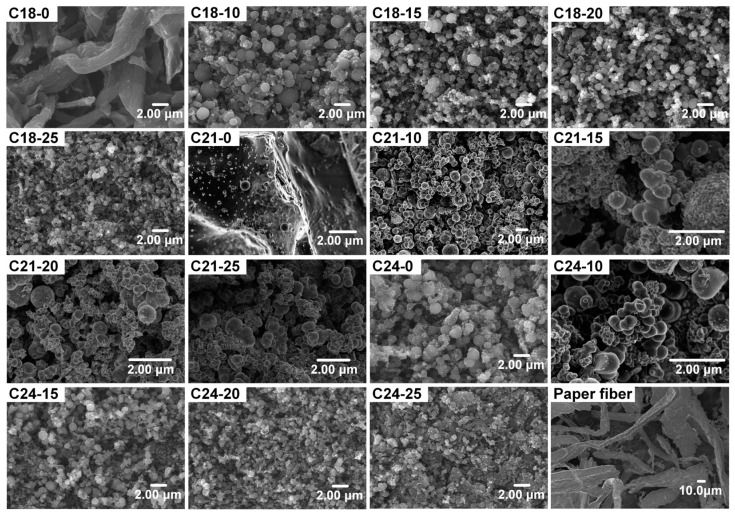
Micromorphology of the carbon microspheres prepared under different conditions.

The micromorphology of the prepared carbon microspheres under different conditions is shown in Figure 1. The results show that the micromorphology of the carbon microspheres is influenced by the reaction temperature and the sulfur acid concentration. The products are not spherical without sulfuric acid at 180 °C and 210 °C, but spherically structured products are obtained at 240 °C without sulfuric acid. When the temperature is 180 °C, the hydrothermal product mostly maintains the original structure of the waste office paper fiber (WOPF). When the temperature rises to 210 °C, most of the product is blocky solid with a smooth surface, and sporadic carbon microspheres occur, which is due to the main component of the WOPF being cellulose. This research shows that cellulose can be hydrolyzed and generates monosaccharides in low-temperature and low-pressure conditions with acid or without catalyst at above 215 °C [27]. Monosaccharides have been used for the synthesis of carbon microspheres under hydrothermal conditions. Since the cellulose in WOPF cannot be hydrolyzed or decomposed without catalyst at 180 °C, the WOPF still keeps its original form after the hydrothermal reaction. When the temperature rises to 210 °C, only a small amount of cellulose or hemicellulose on the surface of the WOPF is hydrolyzed, which makes the surface of the WOPF smooth, and almost no carbon microspheres are produced. However, when the temperature rises to 240 °C, a large amount of carbon microspheres is formed without sulfuric acid. This is because the cellulose in the WOPF is hydrolyzed at this temperature and generates sufficient monosaccharides to form spherical gels through hydrothermal reaction. This also indicates that the critical temperature for cellulose hydrolysis in the absence of catalysts is between 210 °C and 240 °C.

The SEM images show that the average particle size of the prepared carbon microspheres decreases with the increase in sulfuric acid content. There have been reports in the literature that when the acidity increases in a certain range, the hydrolysis reaction of cellulose becomes more complete and the concentration of hydrolysates in the reaction system increases. The higher concentration of monosaccharides increases the frequency of molecular collisions and more sphere cores are formed in the hydrothermal reaction system, which leads to more spheres and a relatively average small particle size. On the other hand, the extreme concentration of sulfuric acid can lead to adhesion between carbon microspheres and non-uniform particle size. In summary, the sulfuric acid concentration and hydrothermal temperature have a combined effect on the granulation rate, particle size and dispersion of carbon microspheres.

### 2.2. FT-IR Analysis

**Figure 2:**
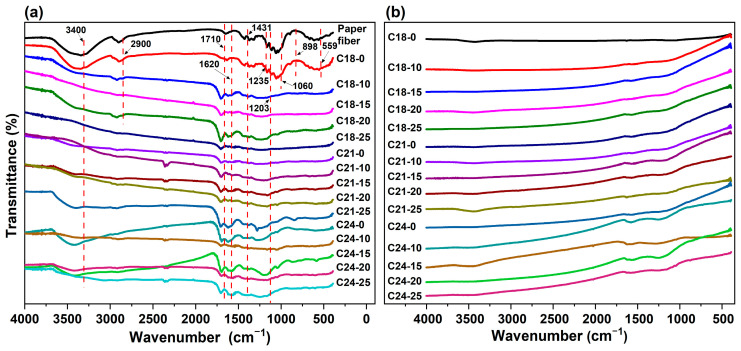
The FT-IR spectra of the waste office paper fiber (**a**), hydrothermal reaction products and (**b**) prepared carbon microsphere.

The FT-IR spectra of the WOPF, obtained hydrothermal reaction products and carbon microspheres are shown in Figure 2. There are five absorption bands in the spectra of the hydrothermal products (Figure 2a). The weak peak in the spectra of the WOPF and C18-0 at 559 cm^−1^ confirms the presence of α-cellulose. The bands at 898 cm^−1^, 1203 cm^−1^ and 1235 cm^−1^ in the spectra of the WOPF and C18-0 also verify the presence of β-cellulose. The strongest infrared bands in the spectra of the WOPF and C18-0 are located near 1060 cm^−1^ and have many weak peaks on both sides, which are due to the ether bond and hydroxyl group in the cellulose molecule. The peak at 1431 cm^−1^ is ascribed to the bending vibration of CH in CH-O. The broad bands at 2900 cm^−1^ and 3000–3700 cm^−1^ are attributed to aliphatic C-H stretching vibration and O-H (hydroxyl or carboxyl groups) [28], respectively. These results show that the cellulose molecule in WOPF cannot be hydrolyzed at 180 °C without sulfuric acid. The characteristic absorption bands of cellulose are also retained in the spectrum of C21-0, which indicates that cellulose in WOPF is not completely hydrolyzed at 210 °C, which is consistent with the SEM results.

There are two main characteristic bands in the spectra of the other samples. The broad absorption band at 1160–1240 cm^−1^ is probably attributable to the C-O-C asymmetrical stretching of fatty acid esters and lactones. The bands at 1710 cm^−1^ and 1620 cm^−1^ probably can be ascribed to C=O (carbonyl, quinone, ester or carboxyl) and C=C vibrations, respectively. The bands at 668 cm^−1^, 2340 cm^−1^ and 2360 cm^−1^ in individual curves are due to carbon dioxide in the atmosphere. All the results show that cellulose in the WOPF can be hydrolyzed when sulfuric acid is added or the temperature is higher than 240 °C. After 24 h of hydrothermal reaction, cellulose-hydrolyzed products are polymerized and carbonized, a and small amount of ether, ester, quinone, carboxyl and other products are also generated. Figure 2b shows that almost all the functional groups of hydrothermal products are removed after 800 °C heat treatment.

### 2.3. X-ray Photoelectron Spectroscopy Analysis

**Figure 3:**
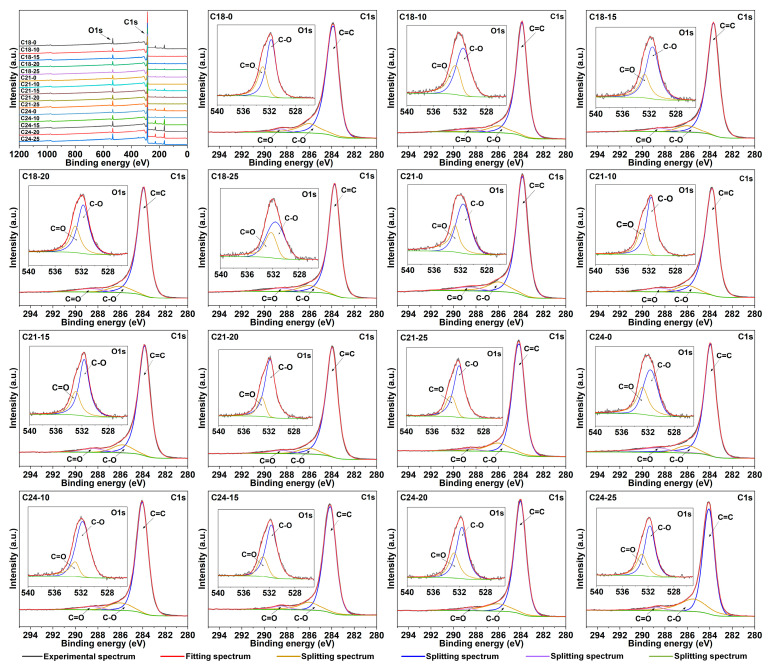
XPS spectra of obtained carbon microspheres.

XPS analysis was carried out on the carbon microspheres to characterize the composition and state of the surface elements. The XPS full spectrum of the carbon microspheres is shown in the first image of Figure 3, and the high-resolution XPS spectra of the elements including C and O in the material are shown in Figure 3. The X-ray photoelectron spectroscopy analysis indicates that the surface of carbon microspheres is composed primarily of 94−98 At.% of C element and less than 6 Atm.% of O element (Table 1).

There are two peaks at the nearby 531.5−532 eV and 533 eV in the high-resolution XPS energy spectrum of O 1s, which correspond to C-O and C=O bonding, respectively. The three peaks at 283.5−284.0 eV, 285.8−287 eV and 287.5−288.5 eV in the spectrum of C 1s correspond to the carbon atoms, C-O and C=O bonding, respectively. The results show that the C element is mainly in the chemical state of C=C bonding, while the O element mainly exhibits the chemical state of C-O and C=O bonding, which is attributed to the incomplete decomposition of the paper fiber. Therefore, the oxygen bond removal method of carbon materials is worthy of further study to make prepared carbon materials with higher carbon content purity.

### 2.4. Thermogravimetric Analysis

**Figure 4:**
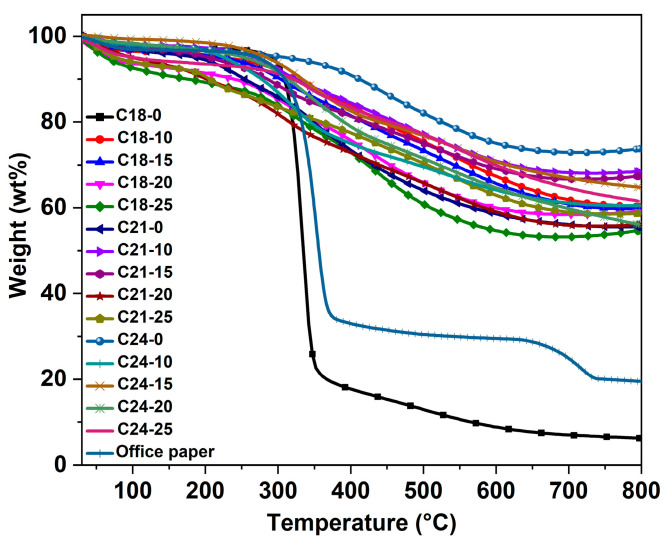
TG curves of the waste office paper fiber and hydrothermal products.

Figure 4 shows the TG curves of the WOPF and the hydrothermal products. It can be seen that a slight weight loss occurred for all the samples from room temperature to 240 °C, which is attributed to the removal of moisture and volatilizable small molecules. In the temperature of 240−600 °C, another weight loss range appears and the thermogravimetric trends of all the hydrothermal products except C18-0 are roughly the same. The weight loss trend of the hydrothermal reaction product (C18-0) without sulfuric acid at 180 °C is similar to that of the raw material, which indicates that the chemical composition does not change obviously after hydrothermal reaction under this condition. This is consistent with the results of FT-IR and confirms that the cellulose is not hydrolyzed. The slight difference between the two curves may be due to the hydrolysis of hemicellulose and the pyrolysis of other substances in the WOPF during the hydrothermal reaction. The weight loss of the WOPF and C18-0 are obvious in the range of 240−370 °C, which is due to the pyrolysis of cellulose and generation of small-molecule gases and large-molecule volatiles. The fracture of ether bonds and ester bonds produces some volatile substances. The obvious weight loss in other samples between 240 and 650 °C is due to the removal of functional groups and the decomposition of organic matter in the hydrothermal products. The office paper used in this study also contains about 27 wt% of other organic and inorganic additives, which are decomposed between 650 and 725 °C.

### 2.5. XRD Analysis

**Figure 5:**
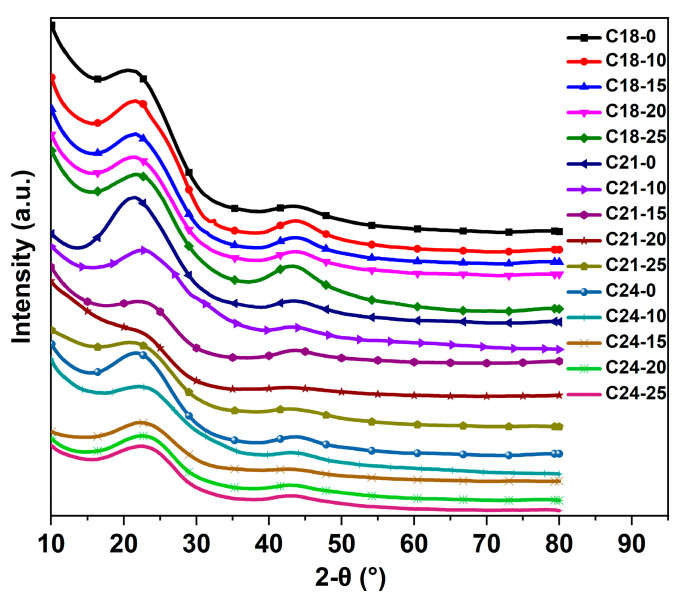
XRD patterns of the prepared carbon microsphere.

Figure 5 shows the XRD patterns of the carbon microsphere. The results show that all samples have two characteristic peaks. A large hump-shaped peak appears near the diffraction angle of 23°, which is the hexagonal lattice diffraction of graphite, indicating that the synthesized carbon microsphere product is composed of amorphous materials. The broader diffraction peak at 43.6° corresponds to the (10) plane [29], which shows that the carbon microspheres have a regular layered structure in the individual layer plane units. The results show that the carbon microsphere products prepared under different reaction conditions have amorphous structure. The crystallite size was calculated via the Debye–Scherrer equation [30] and is shown in Table 2. It can be seen from the figures and tables that the wider the peak, the smaller the corresponding size.

### 2.6. Electrochemical Performance

**Figure 6:**
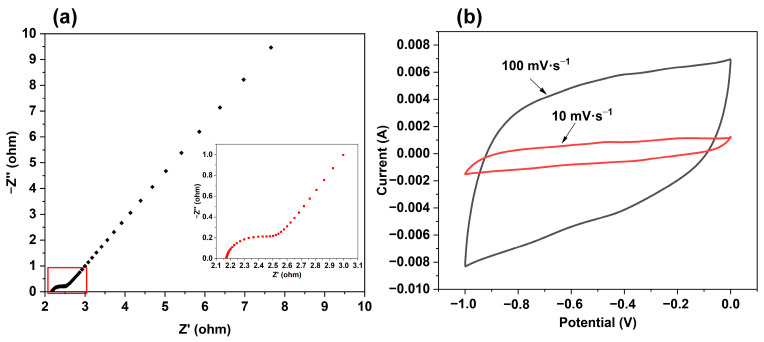
(**a**) Electrochemical impedance spectroscopy diagram and (**b**) cyclic voltammetry curve of the C21−10.

The sample C21-10 with a good morphology was used to analyze the electrochemical performance. Figure 6 shows the electrochemical impedance spectroscopy diagram and cyclic voltammetry curve of the sample C21-10. It can be seen that there is a semicircle in the high-frequency region, and the electrode process is the adsorption process of electrolyte ions on the surface of the electrode material particles. The diameter of the semicircle is lower, and the conductivity of the electrode material is better. The equivalent resistance (ESR) value of 2.17 Ω can be obtained from the intersection of the high-frequency semicircle and the real axis in the figure. The small diameter of the semicircle indicates that the ion transfer resistance is small, which proves that the prepared carbon microspheres have good electrical conductivity. The CV curve of the electrode material C21-10 is similar to a rectangle, and the CV curve can continue to maintain the rectangle shape with the increase in scanning rate, which indicates that the material has a relatively good capacitance performance.

## 3. Formation Mechanism of Carbon Microsphere

**Figure 7:**
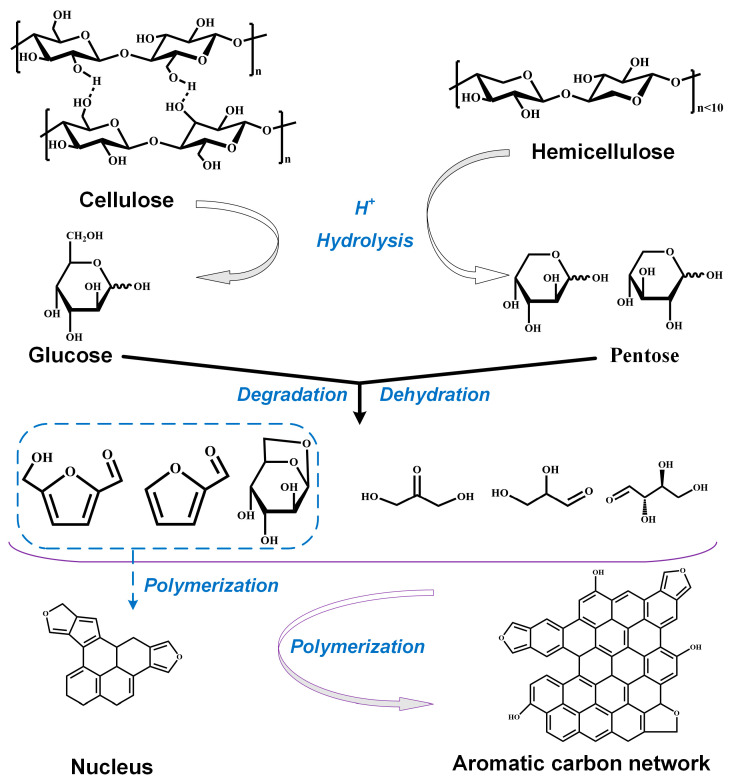
The synthesis reaction mechanism during hydrothermal process.

**Figure 8:**
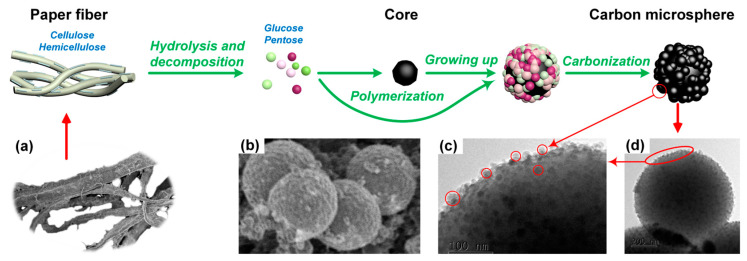
Schematic illustration for the forming process of carbon microspheres. (**a**) SEM image of paper fiber, (**b**) SEM image of carbon microspheres, (**c**) detail of carbon microspheres in TEM image, (**d**) TEM image of carbon microsphere.

The synthesis mechanism of the carbon microspheres is shown in Figure 7 and the schematic illustration for the forming process of the carbon microspheres is shown in Figure 8. Most of the lignin in biomass is removed during the pulp manufacturing process. The cellulose and hemicellulose content of the waste paper used in this study are more than 70 wt%, and the trace amount of lignin can be ignored. In this work, the preparation of carbon microspheres from the WOPF can be summarized into four stages. First, the cellulose and hemicellulose in waste paper are hydrolyzed into monosaccharide. The monosaccharide generated by hydrolysis continues to be held in the hydrothermal condition and chemical reaction occurs. The monosaccharide dehydrates and polymerizes into the intermediate product of 5-hydroxymethylfurfural (HMF). The HMF can form a three-dimension network structure via polycondensation and aromatization reactions, resulting in a large number of core structures. Other hydrolysis and decomposition products in the system polymerize and form plenty of small spherical gels. The amount of the small spheres in the system increase and form larger spheres around the core. The high-temperature treatment under nitrogen conditions removes the oxygen and hydrogen elements of the spherical gel and improves the carbon content of the carbon microsphere final products. In short, the cellulose and hemicellulose in the WOPF must be hydrolyzed to successfully synthesize carbon microspheres.

It was reported that in the hydrothermal carbonization process without catalyst, hemicellulose began to be hydrolyzed at about 180 °C and cellulose at about 210 °C. When the temperature is higher than 220 °C, hydrogen hydrate ions generated by water autoionization catalyze the hydrolysis of cellulose to form oligosaccharides (such as cellobiose, cellobiose, cellobiose, etc.) and glucose [27]. The above conclusions can be verified by the morphology analysis of the final products. The cellulose and hemicellulose in the WOPF cannot be hydrolyzed under the condition of 180 °C without sulfuric acid, resulting in the final product (C18-0) still retaining the original morphology of the WOPF. When the temperature rises to 210 °C, the hemicellulose on the surface of WOPF is hydrolyzed, but cellulose is in the critical hydrolysis state. The TG and FTIR results indicate that there is no cellulose in hydrothermal products and the morphology analysis shows that the final product is a blocky solid with a smooth surface (C21-0). The above results indicate that the cellulose in the WOPF is not completely hydrolyzed into monosaccharides. As a result, the monosaccharide concentration in the system is not enough to form spherical gel, and almost no carbon microspheres are observed in the product. When the temperature reaches 240 °C, hemicellulose and cellulose in the WOPF can be hydrolyzed massively without catalyst. Then the obtained monomers are dehydrated, polymerized and aromatized to synthesize carbon microspheres (C24-0).

Cellulose can be hydrolyzed into glucose under acidic conditions and increasing temperature can accelerate the hydrolysis reaction. Sulfuric acid is most commonly used for the hydrolysis of cellulose. The results indicate that large amounts of carbon microspheres were produced in all samples to which sulfuric acid was added.

## 4. Materials and Methods

### 4.1. Materials

The waste office paper used in this study was from the same batch of office waste printing paper. The components of the waste office paper were analyzed using the Van Soest cellulose determination method and the results are listed in Table 3. The results show that the main components of the waste office paper used in this work are cellulose, hemicellulose and other unknown components such as fillers. The other chemical reagents for the synthesis of carbon microspheres were sulfuric acid (H_2_SO_4_, 98 wt%, Tianjin Kemiou Chemical Reagent Co., Ltd., Tianjin, China), absolute ethyl alcohol (C_2_H_6_O, 99.7 wt%, Tianjin Tianli Chemical, Tianjin, China), deionized water (Produced by UPH series ultra-pure water machine, Ulupure, Chengdu, China), and potassium bromide (KBr, spectral purity, Tianjin Kemiou Chemical Reagent Co., Ltd., Tianjin, China). Sodium hydrogen carbonate (NaHCO_3_, 99.5 wt%, Tianjin Fuchen Chemical Reagents Factory, Tianjin, China) was used for neutralization of waste liquid. Nickel foam (Taiyuan Liyuan Lithium technology Center, Taiyuan, China), acetylene black (Tianjin Youmeng Chemical Technology Co., Ltd., Tianjin, China) and polyvinylidene Fluoride (PVDF, Taiyuan Liyuan Lithium technology Center, Taiyuan, China) were used as the battery grade, and N-methylpyrrolidone (NMP, analytically pure, Sinopharm Chemical Reagent Co., Ltd., Shanghai, China) was used as the solvent. The mercuric oxide electrode (Hg|HgO|OH-, R502, single and double salt bridge universal type) was from Shanghai Yueci Electronic Technology Co., Ltd. (Shanghai, China).

### 4.2. Synthesis of Carbon Microspheres

**Figure 9:**
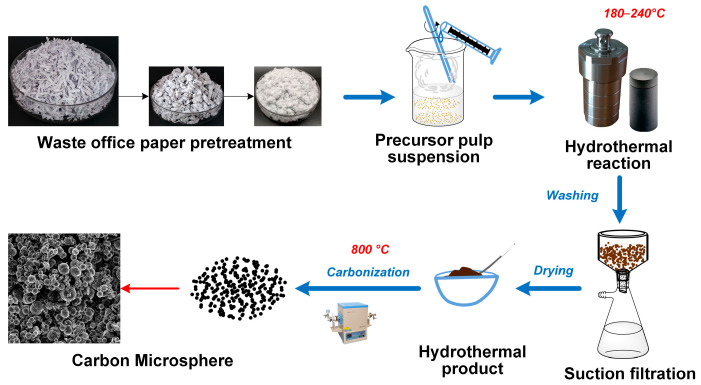
Preparation process of carbon microspheres.

As shown in Figure 9, the WOPF was obtained from waste office paper by beating, drying and grinding, and in these processes some inorganic fillers and additives were removed from the waste paper. Therefore, the main components of the WOPF obtained after simple treatment are cellulose and a small amount of hemicellulose. To ensure the uniformity of the reaction system, the WOPF was dispersed in deionized water and made into pulp by a secondary beating and ultrasonic dispersion for 1 h with stirring. Then, the concentrated sulfuric acid was added into the above paper pulp and stirred for 10 min. The above mixture was transferred into the para-polyphenylene (PPL) liner of a high-temperature and high-pressure reaction kettle and reacted at a certain temperature (180 °C, 210 °C and 240 °C) for 24 h. After the reaction was over, the reactor was cooled to room temperature naturally, and the precipitation was washed and filtered repeatedly with deionized water and anhydrous ethanol until the filtrate was neutral. The obtained products (named gel) were dried at 100 °C for 12 h. The gels were carbonized via tube furnace in a nitrogen atmosphere with a heating rate of 5 °C/min to 350 °C for 1 h, then heated up to 800 °C with a heating rate of 10 °C/min for 2 h. Then, the carbon microspheres was obtained after being cooled down to room temperature naturally. The prepared conditions and corresponding sample number of the carbon microspheres samples are listed in Table 4. The samples were labeled CY-Z, where C is carbon microsphere, Y is the temperature of hydrothermal reaction and Z is the sulfuric acid dosage (the unit is mL). The dosage of sulfuric acid represents the amount of sulfuric acid per 100 mL pulp.

### 4.3. Characterization

The micromorphology and particle size of the obtained carbon microspheres were characterized using field emission scanning electron microscopy (FE-SEM, SU8000, Hitachi, Tokyo, Japan) at 20 kV and high-resolution transmission electron microscopy (TEM, JEM-3010, JOEL, Kyoto, Japan). The diffraction patterns of the carbon microspheres were analyzed using an X-ray diffraction meter (XRD) instrument (XRD-7000, Shimadzu Limited, Kyoto, Japan) at a scanning rate of 5°/min with the 2θ angle varying from 10° to 80°. The functional groups of the gels and carbon microspheres were measured by using a Flourier transform infrared spectroscopy spectrometer (FT-IR, FTIR-8400S, Shimadzu, Kyoto, Japan.) with KBr pellets as sample matrix in the wave number range of 400−4000 cm^−1^ and the scan frequency was 40 time/s. The chemical elements of the obtained carbon microspheres were analyzed via X-ray photoelectron spectroscopy (XPS) (Thermo Scientific K-Alpha+, Thermo Fisher Scientific Inc., Waltham, MA, USA), the vacuum degree of the analysis chamber being 5 × 10^−10^ KPa. The details are as follows: X-ray source: Cu Kα source (*λ =* 1.54056 Å) energy: 1486.6 eV, voltage: 40.0 kV, beam current: 40.0 mA, analyzer scanning mode: continuous scan, instrument work function: 4.2, and the crystallite size was calculated via the Debye–Scherrer Equation (1). Thermogravimetry (TG) was carried out using a thermogravimetric analyzer (TG209 F3, NETZSCH -Gerätebau GmbH, Selb, Germany) in a nitrogen atmosphere from 25 °C to 800 °C at a heating rate of 10 °C/min. The electrochemical performance was analyzed via cyclic voltammetry (CV) with a voltage range of 0−1 V and electrochemical impedance spectroscopy (EIS) with a frequency range of 0.01 Hz−100 kHz on an electrochemical workstation (CHI660E B18120, Shanghai Chenhua Instrument Co., Ltd., Shanghai, China). The carbon microspheres were made into electrodes for testing. The prepared carbon microsphere sample, acetylene black and PVDF were mixed in NMP solvent with a weight ratio of 8:1:1. The mixture was coated on a 1 cm^2^ nickel foam and dried in a vacuum oven for 12 h. The self-made electrode was used as the cathode, Hg|HgO|OH- as the reference electrode, Pt as the counter electrode, and 6 mol/L potassium hydroxide solution was used as the electrolyte to characterize the electrochemical properties of the carbon microsphere.

(1) $$D=\frac{K\lambda }{\beta \cos\theta }$$

*D* is the crystallite size (Å); *K* is Scherrer constant; *β* is the half-height width of the diffraction peak, which needs to be converted into radian (rad) in the calculation process; *θ* is the Bragg angle (rad); and *λ* is the X−ray wavelength [30].

## 5. Conclusions

In this work, carbon microspheres were successfully synthesized from waste office paper via the hydrothermal synthesis method. The main components of the waste paper used in this study are cellulose and hemicellulose, which are more than 70 wt%. The results show that to successfully prepare carbon microspheres, the experimental conditions must ensure that the cellulose in the WOPF can be hydrolyzed. Without sulfuric acid, carbon microspheres can be obtained only at 240 °C, and they can be obtained when sulfuric acid is used at 180 °C 210 °C and 240 °C. The hydrolyzed products (oligosaccharides and monosaccharide) of cellulose and hemicellulose formed spherical gels in hydrothermal conditions by dehydration and polymerization. The carbon microspheres were obtained from the spherical gels via high-temperature treatment. Sulfuric acid concentration and hydrothermal temperature affect the morphology of carbon microspheres by affecting the hydrolysis of cellulose in the WOPF. The granulation rate of the product increased with the increase in sulfuric acid content at the same temperature when the addition of sulfuric acid was less than 10 mL. The granulation rate of carbon microspheres also increased with the increase in hydrothermal temperature when the same amount of sulfuric acid was added. Waste office paper is a good raw material for the preparation of carbon microsphere. The prepared carbon microspheres have good electrical conductivity and capacitance performance. More importantly, they provide a new method for the recycling of waste paper, but controlling the reaction process and the morphology of the carbon microspheres still need further study.

## Figures and Tables

**Table 1:** The relative atomic percentage of chemical state on the surface of carbon microspheres (At.%).

Samples	C	O (C=O)	O (C-O)
C18-0	94.15	1.94	3.91
C18-10	97.27	0.97	1.76
C18-15	96.91	0.95	2.14
C18-20	97.02	0.95	2.03
C18-25	98.00	0.33	1.67
C21-0	97.27	0.76	1.97
C21-10	95.94	1.26	2.80
C21-15	96.19	1.15	2.66
C21-20	96.56	0.78	2.66
C21-25	95.41	1.20	3.39
C24-0	97.31	1.13	1.56
C24-10	96.60	1.00	2.40
C24-15	95.39	1.16	3.45
C24-20	96.52	1.28	2.20
C24-25	94.92	1.58	3.50

**Table 2:** The crystallite size of the diffraction powder (Å).

Samples	D (002)	D (10)
C18-0	29	36
C18-10	28	39
C18-15	33	38
C18-20	24	35
C18-25	25	26
C21-0	16	37
C21-10	21	33
C21-15	24	28
C21-20	19	26
C21-25	18	21
C24-0	20	39
C24-10	21	33
C24-15	17	34
C24-20	18	27
C24-25	16	28

**Table 3:** Components of the waste office paper fiber (wt%).

Components	Cellulose	Hemicellulose	Lignin	Ash-Inorganic Salt	Other
Content	65.247	6.706	0.181	0.676	27.385

**Table 4:** The synthesis conditions of carbon microspheres.

Samples	Temperature (°C)	H_2_SO_4_ (mL)
C18-0	180	0
C18-10	180	10
C18-15	180	15
C18-20	180	20
C18-25	180	25
C21-0	210	0
C21-10	210	10
C21-15	210	15
C21-20	210	20
C21-25	210	25
C24-0	240	0
C24-10	240	10
C24-15	240	15
C24-20	240	20
C24-25	240	25

## Data Availability

Data are contained within the article. The data presented in this study on the synthesis and properties of carbon microspheres from waste office paper are available.

## Sample Availability

Samples of the compounds are not available from the authors.
